# A Cultivated Form of a Red Seaweed (*Chondrus crispus*), Suppresses β-Amyloid-Induced Paralysis in *Caenorhabditis elegans*

**DOI:** 10.3390/md13106407

**Published:** 2015-10-20

**Authors:** Jatinder Singh Sangha, Owen Wally, Arjun H. Banskota, Roumiana Stefanova, Jeff T. Hafting, Alan T. Critchley, Balakrishnan Prithiviraj

**Affiliations:** 1 Department of Environmental Sciences, Faculty of Agriculture, Dalhousie University, PO Box 550, Truro, NS B2N 5E3, Canada; E-Mails: jatinder.sangha@dal.ca (J.S.S.); owenwally@gmail.com (O.W.); 2 Aquatic and Crop Resources Development, National Research Council Canada, 1411 Oxford Street, Halifax, NS B3H 3Z1, Canada; E-Mails: arjun.banskota@nrc-cnrc.gc.ca (A.H.B.); Roumiana.Stefanova@nrc.gc.ca (R.S.); 3 Acadian Seaplants Limited, 30 Brown Avenue, Dartmouth, NS B3B 1X8, Canada; E-Mails: jhafting@acadian.ca (J.T.H.); Alan.Critchley@acadian.ca (A.T.C.)

**Keywords:** β-amyloid, *Caenorhabditis elegans*, cultivated *Chondrus crispus*, glycolipid, monogalactosyl diacylglycerol (MGDG), neuroprotection, red seaweeds

## Abstract

We report here the protective effects of a methanol extract from a cultivated strain of the red seaweed, *Chondrus crispus*, against β-amyloid-induced toxicity, in a transgenic *Caenorhabditis elegans*, expressing human Aβ_1-42_ gene. The methanol extract of *C. crispus* (CCE), delayed β-amyloid-induced paralysis, whereas the water extract (CCW) was not effective. The CCE treatment did not affect the transcript abundance of *amy1*; however, Western blot analysis revealed a significant decrease of Aβ species, as compared to untreated worms. The transcript abundance of stress response genes; *sod3*, *hsp16.2* and *skn1* increased in CCE-treated worms. Bioassay guided fractionation of the CCE yielded a fraction enriched in monogalactosyl diacylglycerols (MGDG) that significantly delayed the onset of β-amyloid-induced paralysis. Taken together, these results suggested that the cultivated strain of *C. crispus*, whilst providing dietary nutritional value, may also have significant protective effects against β-amyloid-induced toxicity in *C. elegans*, partly through reduced β-amyloid species, up-regulation of stress induced genes and reduced accumulation of reactive oxygen species (ROS).

## 1. Introduction

Alzheimer’s disease (AD) is a neurodegenerative disorder amongst humans, which is characterized by loss of memory and cognition, mental depression and early mortality [1]. Though AD is a concern amongst the ageing human population, several recent reports suggest that the disease affects an increasing number of younger people [2,3]. The production of Aβ-oligomers has been identified as a primary cause of neuro-degeneration in AD patients [4]. The toxic effects of Aβ-oligomers are unlikely to decrease in the absence of effective preventive and curative measures. Unfortunately, there is no effective drug in the market for the treatment of AD. Among major AD therapies, reversing Aβ (Aβ_1-42_) toxicity has been the main target for drug development [5]. Aβ-aggregation has also been linked to increased oxidative stress causing neuronal injury and death. It is believed that preventing deposition of Aβ oligomers, reduction of oxidative stresses, or activation of disease modifying pathways could reduce the onset of AD [6,7]. In this regard, it is reasonable to explore the effect of natural compounds from a number of sources to evaluate their effects on the reduction of pathologic markers for AD.

A select number of seaweeds (*i.e.*, red, green and brown) are important components of human diets in several parts of the world, predominantly Southeast Asia. A number of *in vitro* and *in vivo* studies have revealed beneficial health-promoting effects of extracts and compounds isolated from certain seaweeds [8,9]. The wide variety of health-promoting effects of seaweeds is primarily due to their structurally diverse bioactive molecules. For instance, compounds isolated from seaweeds have been shown to possess a wide variety of biological activities, including anti-oxidant, anti-microbial, anti-cancer, anti-inflammatory, anti-coagulant and anti-obesity activities [10]. There is a considerable interest in identifying neuro-protective compounds from seaweeds [11,12]. A large number of seaweed species are yet to be explored for their neuro-protective effects, particularly against β-amyloid toxicity in whole animal studies.

Red seaweeds (Rhodophyta) are known producers of bioactive proteins, sulphated polysaccharides (such as agarans, xylans and carrageenans), vitamins, minerals, pigments and several other components [10,13,14]. However, this group of seaweeds remains a relatively under-utilized resource for mining pharmaceutical, nutraceutical and functional food benefits. A commercial strain of cultivated red seaweed (*Chondrus crispus* or Irish Moss) is used as food and has also been demonstrated to possess significant beneficial bioactivity [15]. *C. crispus* is known to contain several micro and macro-elements, various fatty acids, sterols and polysaccharides such as carrageenans [16]. Recent studies showed the presence of bioactive peptides and prebiotics in selected seaweeds, including *C. crispus*, suggesting their potential health benefits [17,18].

*Caenorhabditis elegans* is a highly suitable animal model for the study of the effects of bioactive components which might have relevance to human health. This is, in part, due to its high level of homology with the human genome [19]. Many of the stress-induced pathways, and their components studied in *C. elegans* are similar to those of humans (e.g., the insulin-like growth factor IGF-1 signal transduction pathway, and the insulin/IGF-1 signal (IIS) transduction pathway regulated SKN-1, an orthologue of human Nrf1Nrf1/2/3 [20]). Moreover, *C. elegans* has a short lifespan and can be cultured with ease in the laboratory. These characteristics make the nematode a convenient model to study biochemical and molecular responses to a variety of environmental stresses. Mechanisms of aging and age-associated neuro-degenerative diseases have been elucidated using *C. elegans* [21]. Transgenic *C. elegans* strains which express human β-amyloid (Aβ), facilitate further enhanced understanding of the mechanisms of Aβ-toxicity in biological systems and can be used in the screening of therapeutic agents *in vivo* [22,23]. Natural products such as extracts from ginkgo leaves (*Ginkgo biloba*), cinnamon bark (*Cinnamomum cassia*), ground coffee seeds (*Coffea* spp.), green tea leaves (*Camellia sinensis*), and others were found to reduce Aβ-induced paralysis in *C. elegans*. Furthermore, using this model, the molecular mechanism of activity of these products was also revealed [1,23,24].

In this study, we investigated the effects of chemical components from a cultivated strain of the red seaweed *C. crispus* against Aβ-induced toxicity using a transgenic *C. elegans* model for AD. Bioassay-guided fractionation of an organic extract of *C. crispus* resulted in identification of lead molecules which protected *C. elegans* against Aβ-toxicity. The molecular mechanism(s) of biological activity of these compounds are also discussed.

## 2. Results

### 2.1. CCE Alleviated Aβ-Induced Paralysis in C. elegans

**Figure 1:**
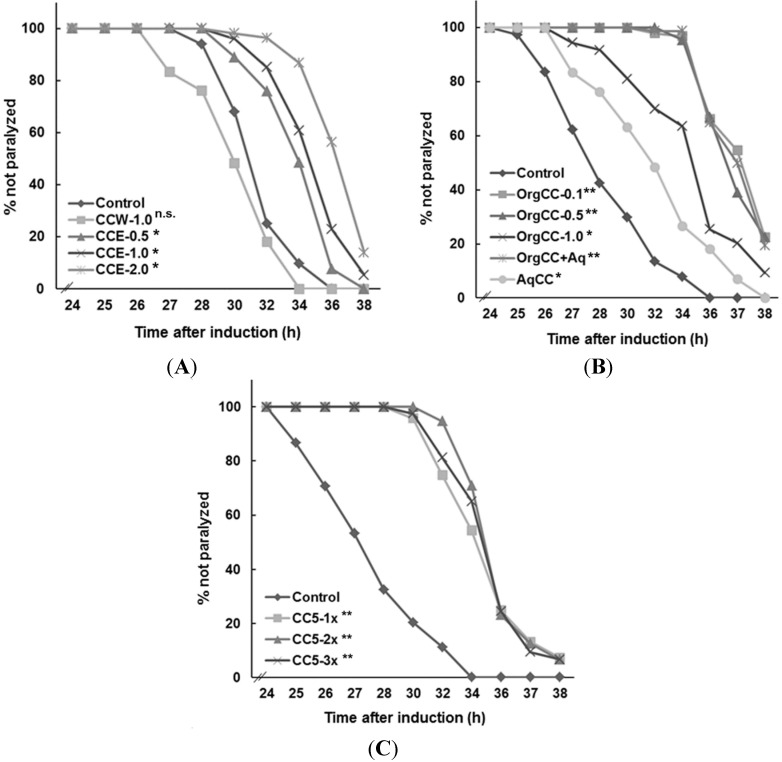
*C. crispus* delayed Aβ induced paralysis of transgenic *C. elegans* strain CL4176 expressing human β-amyloid gene. The worms were healthy at 16 °C but showed paralysis phenotype 25 h after temperature up-shift to 23 °C. *C. elegans* paralysis was expressed as percent based on different experiments (*N* = 180). (**A**) Aβ-induced paralysis was delayed in *C. crispus* methanol extract (CCE) treatments compared to control (NGM only) and *C. crispus* water extract (CCW); (**B**) The organic fractions of CCE (OrgCC) protected the worm against Aβ toxicity when compared to control (**C**) The *C. crispus* sub fraction CC5 significantly delayed Aβ-induced paralysis in *C. elegans* * *p* < 0.05, ** *p* < 0.0001, n.s. = not significant.

CCE (*i.e.*, *Chondrus crispus* methanol extract) was significantly more effective, as compared to the water extract, in protecting *C.elegans* against Aβ-induced toxicity (Figure 1). The onset of Aβ-induced paralysis was significantly delayed with CCE (*p* < 0.05) treatment, at a concentrations of 0.5–2.0 mg/mL, as compared to the control (Figure 1A). In the control, worms exhibited the paralysis phenotype at 25 h onwards and the majority of the worms were paralyzed by 32 h, after the temperature up-shift (Figure 1A). In CCE treatment, the onset of paralysis was delayed until after 28 h and a considerable number of worms were not paralyzed until 36 h. CCW (*i.e.*, *Chondrus crispus* water extract, 1 mg/mL NGM) did not protect the worms against Aβ-toxicity and all the worms were paralyzed similar to the control. These observations suggested that *C. crispus* and, more specifically, the components in CCE have the potential to protect against Aβ-induced toxicity.

### 2.2. Efficacy of CCE Fractions in Protecting C. elegans against Aβ-Induced Toxicity

CCE with significant protective effect against Aβ-toxicity was further fractionated by liquid/liquid partitioning into aqueous (AqCC) and organic (OrgCC) fractions. The OrgCC yield was 1/10th of the methanol extract. Therefore, 0.1 mg of OrgCC was used as an equivalent to 1 mg of CCE. Bioassays with the CL4176 worms revealed a trend similar to that with the CCE; all concentrations of OrgCC tested (0.1–1.0 mg/mL) delayed the onset of Aβ-induced paralysis (Figure 1B). The AqCC fraction also showed some level of protection as compared to control, but the effect was not as prominent as the OrgCC fraction. The organic extract was then fractionated into seven sub-fractions (CC1–CC7), each of which was tested for activities against Aβ-toxicity in transgenic *C. elegans*. Sub-fractions CC1, CC2, CC5 and CC6 performed better in protecting the worms, by delaying the onset of the paralysis phenotype (data not shown). However, the activity of sub-fraction 5 (CC5) showed a significantly higher activity than the rest of the sub-fractions and was tested at different concentrations against *C. elegans* (Figure 1C).

### 2.3. CCE Treatment Reduced the Accumulation of Aβ Peptide in C. elegans

**Figure 2:**
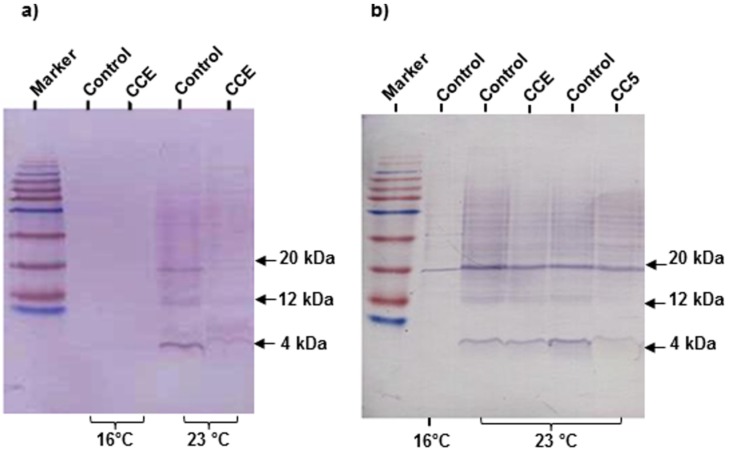
CCE reduced β-amyloid species in *C. elegans*. (**a**) Western blot of Aβ species in the transgenic *C. elegans* CL4176 with and without CCE treatment. Lanes 1 and 2 were loaded with CL4176 proteins from 16 °C whereas lane 3 (MeOH control) and lane 4 (CCE) were loaded with protein extracted from worms incubated in permissive temperature (23 °C); (**b**) Western blot of Aβ species in the transgenic *C. elegans* CL4176 with CCE and fraction CC5 as compared to untreated controls. Lanes 1 was loaded with CL4176 proteins from 16 °C, whereas lanes 2 and 4 (MeOH control), lane 3 (CCE) and lane 5 (CC5) were loaded with protein extracted from worms incubated in permissive temperature (23 °C). The proteins were loaded on 16% acrylamide gel and immuno-blotted with an anti-Aβ antibody (6E10).

To determine if the delayed onset of paralysis with the CCE treatment was associated with decreased Aβ accumulation in transgenic CL4176, Western blot analyses of CCE-treated worms sampled at 25 h after the temperature upshift (Figure 2a) was performed. A visual observation of the immuno-blot developed with anti-Aβ monoclonal antibody 6E10, showed significant differences in monomeric and oligomeric Aβ species in control and CCE treated samples after temperature upshift. The quantitative analysis of Aβ bands with ImageJ showed a reduction in Aβ proteins (4 and 20 kD) in three independent experiments and on an average, more than 30% reduction in Aβ accumulation was observed. The Aβ species were also detected in transgenic worms treated with sub-fraction (CC5) of *C. crispus*. The immunoblotting analysis revealed a similar trend with the fraction CC5 as Aβ species were lower than in the untreated worms (Figure 2b).

### 2.4. The Effect of CCE on the Expression of Aβ and Stress Response Genes in C. elegans

**Figure 3:**
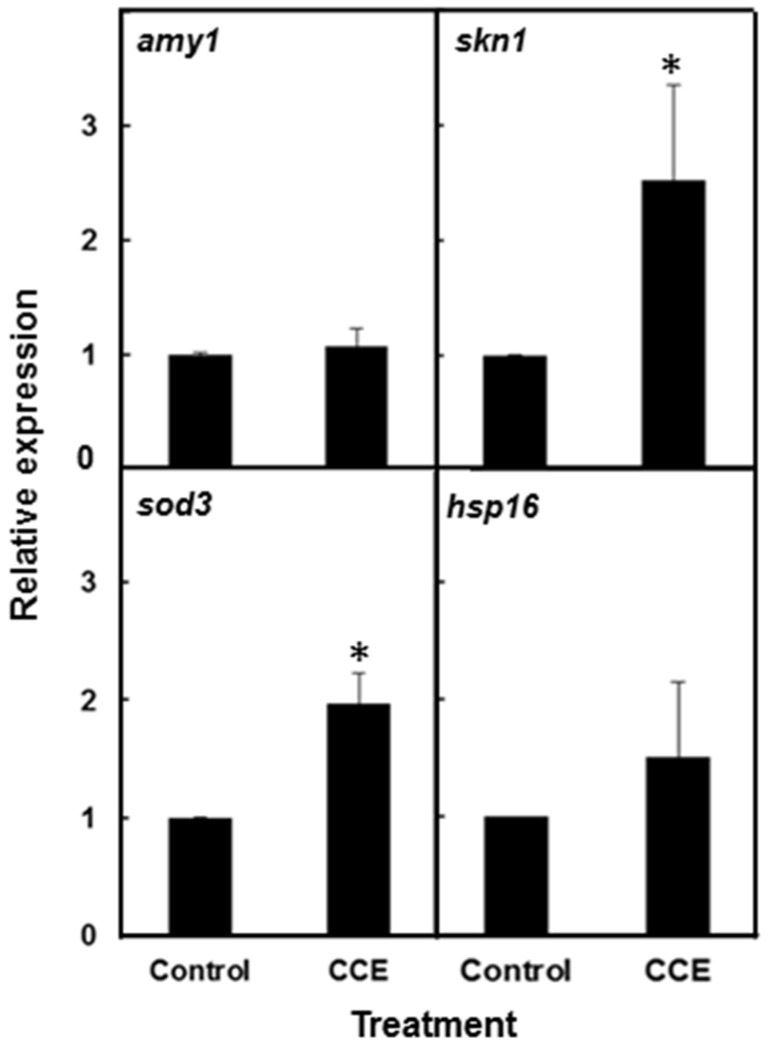
CCE altered expression of stress responsive genes in *C. elegans*. Quantitative real-time PCR was performed using CL4176 worms with or without CCE treatment to quantify the expression of *amy1*, *sod3*, *hsp16.2* and *skn-1* genes. *C. elegans* were raised at 16 °C and then shifted after 36 h to 23 °C. The worms (~80–100) were removed at 20 h after the temperature up-shift for RNA extraction. The relative gene expression was determined using 2^−ΔΔCt^ method. The gene *amy-1* was used as the internal control. Data represent an average from two independent experiments (* *p* < 0.05).

To determine if CCE treatment affected the expression of the Aβ gene, the Aβ mRNA levels were quantified with Real Time RT-PCR. Both CCE-treated and control worms harvested 20 h after the temperature up-shift, did not show any significant difference in Aβ-transcript levels (Figure 3). In order to study the effect of CCE on the expression of stress induced genes that might contribute to protection against Aβ-toxicity, transcript abundance of *hsp-16.2*, *sod-3* and *skn-1* was quantified. The results showed that transcripts of *hsp-16.2*, *sod-3* and *skn1* expressed by 1.4–2.5 fold in CCE treated worms, as compared to the control. The expression of *hsp-16.2* showed only a small increase (1.4 fold) whereas the transcript of *skn-1*, a transcriptional response factor in oxidative stress, was increased by 2.5 fold.

### 2.5. CCE Reduced Toxic Reactive Oxygen Species (ROS) in Transgenic C. elegans

**Figure 4:**
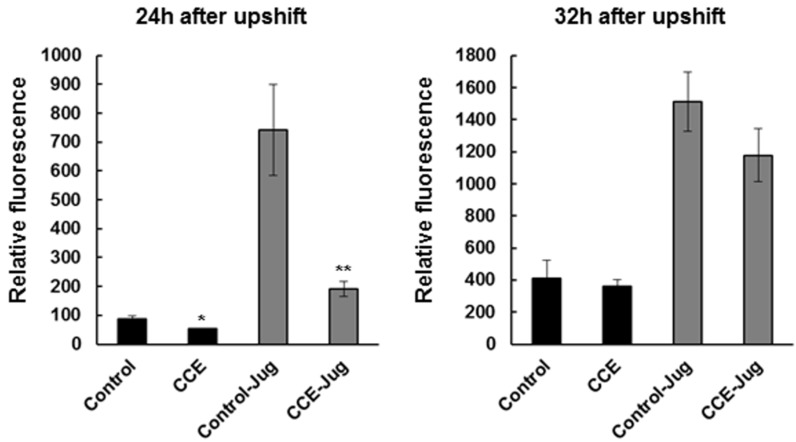
CCE attenuated oxidative stress in *C. elegans*. ROS were measured in CCE and untreated CL4176 worms with or without 100 µM Juglone at 24 and 32 h after temperature upshift. The ROS in worms was measured using 2′,7′-dichlorofluorescein diacetate with 0 or 300 µm juglone. Results are expressed as DCF (2′,7′-dichlorofluorescein) fluorescence relative to the untreated control. Data represent mean ± SE, (*N* = 180) (* *p* < 0.05, ** *p* < 0.001).

The CL4176 worms treated with CCE showed lower levels of ROS *in vivo* measured using the H2DCF-DA (2′,7′-dichlorodihydrofluorescein diacetate) method (Figure 4). The mean fluorescence in transgenic worms at 24 h after temperature upshift was significantly reduced (*p* < 0.05) with CCE treatment than in the control (MeOH) worms. This effect was also evident with addition of juglone used as additional stress to the worms. This trend was same at 32 h after temperature upshift, although the differences were not significant.

### 2.6. Visualization of GFP Tagged Gene Expression in CCE Treated C. elegans

**Figure 5:**
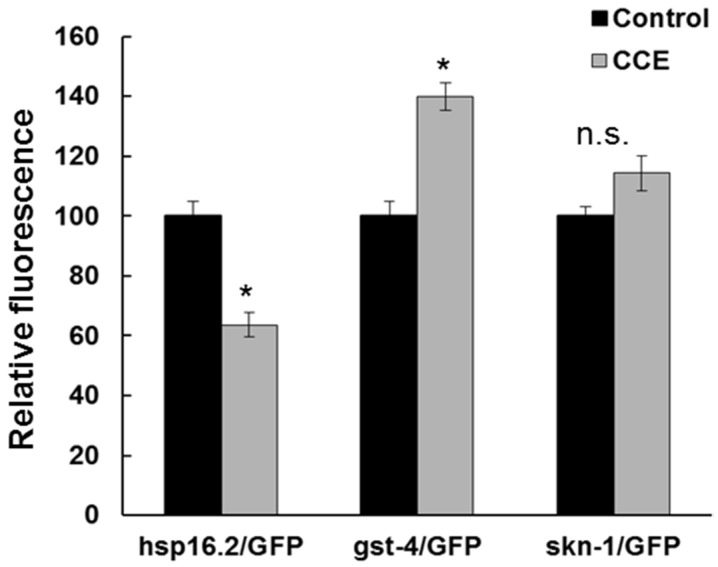
Visualization of GFP tagged genes in *C. elegans*. Expression of *hsp-16.2*, *gst-4*, and *skn-1* tagged GFP reporter in *C. elegans* was visualized using fluorescence microscopy. The images were used to quantify fluorescence at 72 h using ImageJ software (National Institutes of Health, Bethesda, MD, USA). Data are expressed as GFP intensity (mean SE) relative to control obtained from two independent experiments with at least 15 worms in each experimental group (*N* = 30, * *p* < 0.05).

The effect of CCE on stress response genes in *C. elegans* was also determined using transgenic *C. elegans* strains with GFP reporter tagged with *hsp-16.2*, *gst-4*, and *skn-1*. The *hsp-16.2*/*GFP* expression in TJ375 strain was quantified in the head region. Interestingly, the fluorescence was remarkably reduced in the treatment as compared to the untreated worms (*n* = total of 15 worms, *p* < 0.05, Figure 5). The results suggested that CCE treated worms experienced reduced stress resulting in a lower level of *hsp-16.2*/*GFP* fluorescence. The expression of *gst-4*/GFP was observed in transgenic strain CL2166. The CCE exposure resulted in a 40% increase in GFP fluorescence, as compared to the control (Figure 5). The expression of *skn-1*/GFP was investigated in the nuclei of the *C. elegans* strain LD1. CCE treatment caused translocation of *skn-1*/GFP to the intestinal nuclei, under oxidative stress (data not shown), and that the GFP intensity was higher than the control, although the difference was not significant (Figure 5).

### 2.7. Chemical Analysis Revealed the Presence of Glycolipids in CCE

**Figure 6:**
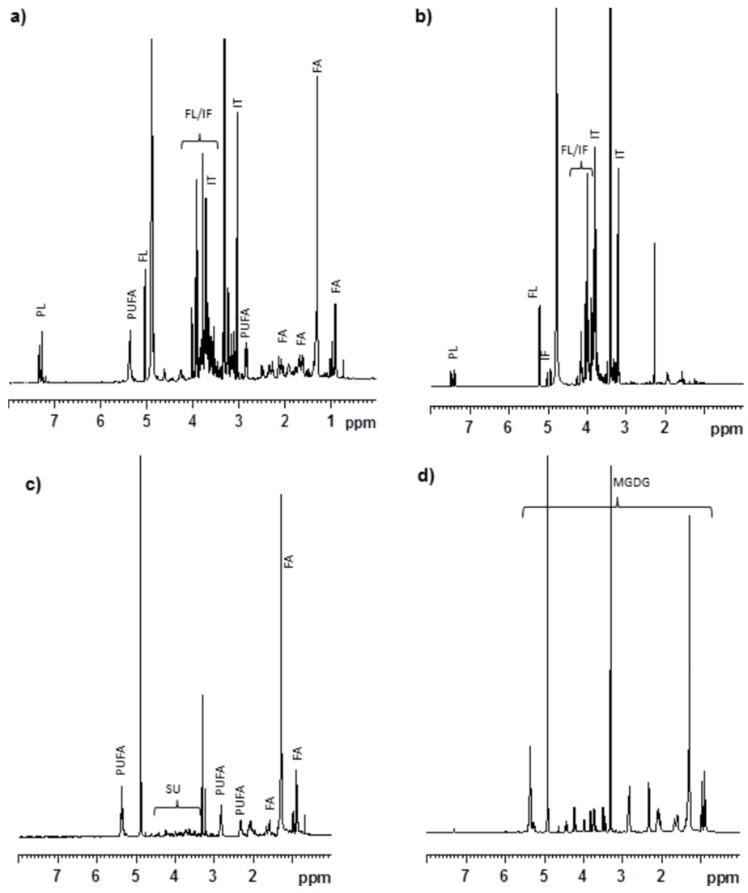
Chemical analysis of *C. crispus* extracts. The ^1^H NMR spectra. (**a**) MeOH extract (CCE); (**b**) Aqueous soluble part of MeOH extract (AqCC); (**c**) Organic soluble part of MeOH extract (OrgCC); (**d**) The sub-fraction 5 (CC5). Signal corresponds to PUFA—polyunsaturated fatty acid, FA—fatty acid, SU—sugar, PL—phenylalanine, FL—floridoside, IF—isofloridoside, IT—isethionic acid, MGDG—mono galactosyl diacylglycerols.

The ^1^H NMR spectrum of CCE gave signals which correspond to floridoside, phenylalanine, isethionic acid, unsaturated fatty acids and photosynthetic pigments (Figure 6a). The ^1^H NMR spectrum of the aqueous fraction of the MeOH extract (AqCC) had signals which primarily corresponded to floridoside, phenylalanine, and isethionic acid (Figure 6b). The concentrations of major metabolites present in CCW, CCE and AqCC were determined by ^1^H NMR spectral analysis and described in Table 1. The results indicated that CCE contain 9.08% isethionic acid followed by floridoside 7.51% and phenylalanine 0.74%. OrgCC which covers 1/10th of CCE showed signals corresponding to polyunsaturated fatty acids, sugars and glycerol (Figure 6c). In our earlier report, we have isolated and identified lutein, ecosapentanoic acid (EPA), aracadonic acid (AA), MGDG and DGDG in the OrgCC fraction [25]. Moreover, the ^1^H NMR spectrum of the most active sub-fraction (CC5; cover 5.55% of the OrgCC fraction by weight) had signals mainly corresponding to monogalactosyldiacylglycerols (MGDG; Figure 6d). Based on the LC/MS data the major MGDG present in sub-fraction CC5 were identified as (2*S*)-1,2-*bis*-*O*-eicosapentaenoyl-3-*O*-β-d-galactopyranosylglycerol, (2*S*)-1-*O*-eicosapentaenoyl-2-*O*-arachidonoyl-3-*O*-β-d-galactopyranosylglycerol, (2*S*)-1-*O*-(6*Z*,9*Z*,12*Z*,15*Z*-octadecatetranoyl)-2-*O*-palmitoyl-3-*O*-β-d-galactopyranosylglycerol, (2*S*)-1-*O*-eicosapentaenoyl-2-*O*-palmitoyl-3-*O*-β-d-galactopyranosylglycerol, (2*S*)-1,2-*bis*-*O*-arachidonoyl-3-*O*-β-d-galactopyranosylglycerol and (2*S*)-1-*O*-arachidonoyl-2-*O*-palmitoyl-3-*O*-β-d-galactopyranosylglycerol. These MGDG cover 71.2% of sub-fraction CC5 based HPLC chromatogram, among them the percentage of the first three MGDG were 20.0, 14.5 and 20.5%, respectively. (2*S*)-1-*O*-Eicosapentaenoyl-2-*O*-palmitoyl-3-*O*-β-d-galactopyranosylglycerol and (2*S*)-1,2-*bis*-*O*-arachidonoyl-3-*O*-β-d-galactopyranosylglycerol signals were overlap in HPLC and covers 10.1%, while (2*S*)-1-*O*-arachidonoyl-2-*O*-palmitoyl-3-*O*-β-d-galactopyranosylglycerol account for 6.0% of the fraction CC5. The polar fractions eluted with CHCl_3_/MeOH (9:1) and MeOH (*i.e.*, sub-fractions 6 and 7) contained a mixture of MGDG and digalactosyldiacylglycerols (DGDG) and because of their complexity and less biological potency, no quantitative analysis was performed for these sub-fractions.

**Table 1:** Concentration of major metabolites in water extract (CCW), MeOH extract (CCE) and aqueous soluble part of MeOH extract (AqCC).

Compound	Percentage (%) in Water Extract (CCW)	Percentage (%) in MeOH Extract (CCE)	Percentage (%) in Aqueous Soluble Part of MeOH Extract (AqCC)
Floridoside	17.68	7.51	8.11
Isofloridoside	*	*	0.81
Isethionic acid	12.02	9.08	9.10
Phenylalanine	0.86	0.74	0.79

The concentrations of individual metabolite were calculated based on integral value of ^1^H NMR signal as compare sodium-(trimythylsilyl)-2,2′,3,3′-tetradeuratedpropionate (TMSP) signal used as an internal standard. * Unable to calculate because of anomeric proton of isofloridoside overlap with water signal.

## 3. Discussion

Neurodegenerative Alzheimer’s disease (AD) largely associated with ageing has been difficult to treat, as information on etiology of the disease is not clear. Nutritionally rich food has been proposed to mitigate neurological diseases such as AD [26]. Seaweeds are being explored for their neuro-protective potential, particularly due to the presence of abundant and diverse bioactive components, which are reported to impart numerous animal and human health benefits. In this paper, we evaluated the effects of extracts from a commercially cultivated strain of the red seaweed, *C. crispus* on Aβ-induced toxicity using a transgenic model *C. elegans* expressing human Aβ_1-42_. We demonstrated that organic components of the seaweed, in particular glycolipids, delayed the onset of β-amyloid-induced paralysis, which, in turn, reduced low molecular weight Aβ species in the worm, and enhanced the transcript abundance of a number of stress-induced genes, which subsequently reduced ROS levels in the treated *C. elegans*.

This study investigated whether the chemical components present in *C. crispus* extracts offered protective effects against Aβ-toxicity in *C. elegans* expressing human Aβ_1-42_ [4]. The methanol extract of *C. crispus* delayed the paralysis induced by Aβ-toxicity, while the water extract was not effective. Bioassay guided fractionation of the methanol extract was effective in delaying the onset of Aβ-amyloid-induced paralysis in *C. elegans* strain CL4176. However, this fraction did not suppress *amy1* transcript abundance in the nematode, thereby suggesting that the biological effect of the chemical component of the fraction was acting at a post-transcriptional level. An examination of Aβ in *C. elegans* strain CL4176 revealed that the concentrations of Aβ species were reduced in the treatment as compared to the control. A similar decrease in the Aβ species has been reported in response to treatment with natural products while the *amy-1* transcripts were not affected or reduced [1]. However, CCE did not reverse the paralysis phenotype caused by deposition of Aβ plaques. The results suggested that the CCE-mediated protection against Aβ toxicity was, to some extent, through a reduction in the Aβ plaque deposition and lower amyloid species in the worms.

AD pathogenesis, specifically Aβ plaques, leads to oxidative stress which is associated with ROS accumulation that can further aggravate the pathological condition in AD patients [27]. We observed here that CCE treatment reduced ROS species in the worms expressing Aβ (CL4176), which might have been either due to the reduction in the formation of toxic Aβ species or induction of antioxidant systems in the worm, thereby reducing the negative effects of Aβ. Antioxidant therapies have been suggested as a potential route to mitigate pathology associated AD [28]. Earlier work suggested that *C. crispus* extracts had mild anti-oxidative potential [15]. In N2 wild type, treatment with a methanol extract of *C. crispus* showed a strong protection against Juglone-induced oxidative stress [15]. Natural products have been shown to reduce ROS, and are also shown to be neuro-protective in *C. elegans*. Our results support the hypothesis that the ROS reducing activity of CCE might be one of the mechanisms by which it protected *C. elegans* against Aβ toxicity. The induction of superoxide dismutase 3 (*sod-3*) transcript in the *C. elegans* strain CL4176, with CCE clearly demonstrated that antioxidant systems were activated in the treated worms and that might have acted to protect the worms against Aβ induced stress. Induction of small heat-shock proteins HSP-16 by Aβ was also reported to offer protection against *in vivo* Aβ peptide toxicity in *C. elegans* [29]. However, we observed that CCE treatment caused only a small increase in *hsp-16.2* transcript. Surprisingly, the *hsp-16.2*/GFP fluorescence intensity in CCE treatment was lower than in the control worms. Thus, it appears *hsp-16.2* had a marginal role in CCE-mediated tolerance to Aβ toxicity in the nematode and shifted the focus of this study towards other mechanisms perhaps functioning to enhance plaque clearance and modulate oligomerization of Aβ.

It appears that SKN-1 in *C. elegans* was affected with CCE treatment. First of all, CCE treatment increased the expression of the *gst-4*/GFP which is regulated by the SKN-1 transcription factor. Gene expression results also showed increased expression of *skn-1*. Further, induction of *skn*-1/GFP was enhanced in transgenic worms with CCE treatment. This evidence, taken together, suggests that the protection from Aβ toxicity in *C. elegans* could have resulted from the downstream induction of effectors in the SKN-1 pathway, which might function as antioxidants [1]. SKN-1 is a functionally analogous to Nrf2 [1], which is known to be involved in phase II detoxification response genes and also in cellular responses to oxidative stress. SKN-1 might be acting in a way similar to Nrf2, with CCE treatment. The induced response of Nrf2 was reported to protect an amyloid precursor protein/presenilin-1 (APP/PS1) transgenic mouse model [30] and neuronal cells, exposed to exogenous Aβ. In *C. elegans*, similar results were reported by Dostal *et al.*, [1], attributing the role of *skn-1* in coffee extract-mediated protection, against Aβ toxicity. These findings suggest that the role of CCE-mediated protection against Aβ-toxicity is at least in part, through the SKN-1 pathway in *C. elegans*.

In an earlier study, we reported that the MeOH extract of *C. crispus* contained mainly floridoside, isethionic acid, taurine, phenylalanine and other lipophilic metabolites including polyunsaturated fatty acids [15]. Further ^1^H NMR study suggested that the organic soluble portion (*i.e.*, OrgCC) had signals corresponding to polyunsaturated fatty acids, and other lipids which would have shown protective activity against Aβ toxicity. The AqCC on the other hand contained primarily floridoside, isethionic acid and phenylalanine, which displayed no protective activities. Sub-fraction 5 (CC5) eluted with CHCl_3_/MeOH (9:1), that exhibited the most protective activity against Aβ-toxicity in *C. elegans*, and contained predominantly MGDG (Figure 5C), were identified as (2*S*)-1,2-*bis*-*O*-eicosapentaenoyl-3-*O*-β-d-galactopyranosylglycerol, (2*S*)-1-*O*-eicosapentaenoyl-2-*O*-arachidonoyl-3-*O*-β-d-galactopyranosylglycerol, (2*S*)-1-*O*-(6*Z*,9*Z*,12*Z*,15*Z*-octadecatetranoyl)-2-*O*-palmitoyl-3-*O*-β-d-galactopyranosylglycerol, (2*S*)-1-*O*-eicosapentaenoyl-2-*O*-palmitoyl-3-*O*-β-d-galactopyranosylglycerol, (2*S*)-1,2-*bis*-*O*-arachidonoyl-3-*O*-β-d-galactopyranosylglycerol and (2*S*)-1-*O*-arachidonoyl-2-*O*-palmitoyl-3-*O*-β-d-galactopyranosylglycerol [25] and accounts for over 71.2% of the fraction. Lipid-containing seaweed extract fractions are known to have an abundance of polyunsaturated fatty acids (PUFAs), glycolipids and unique blends of pigments. Glycolipids constitute an important class of membrane lipids in red seaweeds and possess a broad spectrum of biological activities. Even though water-soluble components such as sulphated polysaccharides, floridoside and iso-floridoside have properties such as cell protection [31,32], we did not observe any positive effect against Aβ-toxicity in *C. elegans* by CCW whereas AqCC showed only a mild effect in delaying the paralysis phenotype. When tested, purified water soluble components were not effective against Aβ toxicity (data not shown). These results supported the conclusion that the lipid rich portion of the MeOH extract of the commercially cultivated *C. crispus* was responsible for the protective effects against Aβ-amyloid toxicity in *C. elegans*. The MGDG-rich fraction was the most active class of lipids present in the OrgCC suggesting that the bioactivity of the *C. crispus* extracts was due to this special class of lipids. MGDG and DGDG are major constituents of chloroplast lipids of plants and algae, it will be interesting to investigate if MGDGs from plants also alleviate Aβ related toxicity in *C. elegans*, similar to the effect as observed with MGDG enriched fractions of *C. crispus*.

In conclusion, CCE has protective effects against β-amyloid toxicity in *C. elegans* through reduced deposition of amyloid species, increased antioxidant activity, and the activated SKN1 pathway in the transgenic worm model. The bioactivity of CCE was primarily attributed to the MGDG-enriched fraction of the extract. The results also suggest the further need to investigate whether these health benefits are observed with dietary *C. crispus* in animal models.

## 4. Materials and Methods

### 4.1. Chemicals

H2DCF-DA (2′,7′-dichlorodihydrofluorescein diacetate), Juglone (5-hydroxy-1,4-naphthoquinone), MGDG, quercetin, secondary anti-mouse IgG alkaline phosphatase conjugate and SigmaFast™ BCIP^®^/NBT tablets were purchased from Sigma-Aldrich (St. Louis, MO, USA). Bradford Reagent was purchased from Biorad (Mississauga, ON, Canada), secondary alkaline phosphatase conjugate and amyloid antibody 6E10 was purchased from Covance (Montreal, QC, Canada). *G. biloba* extract (EGb761) was a kind gift from Dr. Willmar Schwabe Pharmaceuticals, Germany.

### 4.2. Nematode Strains

The wild-type *C. elegans* strain N2 (Bristol) and transgenic worms, TJ375 (*hsp-16.2*/GFP), CL2166 (*gst-4*/GFP) CL2166 (dvIs19[pAF15(gst-4::GFP::NLS)]), LD1 (*skn-1*/GFP), and CL4176 (smg-1ts [*myo-3*/Aβ1–42 long 3′-untranslated region (UTR)]) were purchased from the *Caenorhabditis* Genetics Center (University of Minnesota, Minneapolis, MN, USA). The transgenic nematode strains CL4176 expressed muscle-specific Aβ_1–42_ [4] leading to a paralysis phenotype of the worm under non-permissive conditions. All *C. elegans* strains were maintained at 20 °C, except strain CL4176, which was maintained at 16 °C, on solid nematode growth medium (NGM), seeded with live *E. coli* (OP50) as a food source.

### 4.3. Seaweed Extraction and the Preparation of NGM

Both water (CCW) and methanol (CCE) extracts were prepared from a commercially cultivated strain of the red seaweed *C. crispus* [33]. The strain is proprietary and owned by the National Reseach Council (NRC) of Canada, and exclusively cultivated in Nova Scotia by Acadian Seaplants Limited. This strain of *C. crispus* is cultivated in the world’s largest on-land cultivation facility for the Asian food market (marketed as Hana-Tsunomata™). A voucher specimen of the sample is kept at the Marine Bioproducts Laboratory, Department of Environmental Sciences, Dalhousie University, Canada. The *C. crispus* was collected from production tanks, rinsed with distilled water and immediately lyophilized and vacuum sealed. For preparation of the extracts, the lyophilized seaweed (1000 g) was extracted with Methanol (MeOH) (5L × 3), stirring at room temperature for 1 h, followed by sonication for 15 min. The combined MeOH extract was evaporated under reduced pressure to yield *C. crispus* MeOH extract (CCE). The MeOH extract (50 g) was suspended in water (600 mL) and extracted with ethyl acetate (EtOAc) (600 mL × 2). Both aqueous (AqCC) and organic (OrgCC) extracts were dried under reduced pressure and lyophilized yielding 45.0 g and 5.0 g, respectively. OrgCC (4.0 g) was further fractionated by solid phase extraction, using a silica gel cartridge (Strata SI-1, 70 g, Phenomenex, Torrance, CA, USA) into 7 sub-fractions, eluting with hexane/EtoAc/CHCl_3_/MeOH gradients [sub-fraction 1, hexane/EtOAc (3:1), 284 mg; sub-fraction 2, hexane/EtOAc (1:1), 678 mg; sub-fraction 3, hexane/EtOAc (1:1), 106 mg; sub-fraction 4, EtOAc, 18 mg; sub-fraction 5, CHCl_3_/MeOH (9:1), 222 mg; sub-fraction 6, CHCl_3_/MeOH (1:1); 925 mg; sub-fraction 7, MeOH, 1035 mg]. The seaweed powder (10 g), in a separate process, was extracted with boiling water (30 min each, total water 200 mL), water was then removed at reduced pressure and dried at 70 °C to yield the water extract (CCW).

The seaweed extracts were mixed in nematode growth medium (NGM) to a final range of concentrations form 0.1 to 2.0 mg/mL of NGM using 0.05%–0.1% methanol, just before dispensing in Petri plates. The organic sub-fractions, or pure compounds, were added to NGM as 1 mg equivalent. To inhibit microbial contamination, gentamycin sulfate was added to the NGM at a concentration of 30 µg/mL. The *E. coli* OP50, spread on to the NGM, served as food for *C. elegans*. Plain NGM or NGM + MeOH served as the controls. An extract of *G. biloba* (EGb761; 0.1–1 mg/mL NGM), and in some experiments, quercetin (200 µg/mL NGM) were also used for comparison.

### 4.4. Bioassays for β-Amyloid Induced Paralysis

To determine if *C. crispus* extracts suppressed or delayed the onset of β-amyloid-induced, progressive paralysis of the *C. elegans* strain CL4176, expressing muscle-specific Aβ_1–42_ [4], synchronized eggs were transferred onto Petri dishes containing solidified NGM, either mixed with extract or the controls (water or MeOH). Paralysis was induced in the worms following the method described in Sangha *et al.* [34]. Worms were scored as paralyzed if they failed to move their body or moved only the head when touched with a platinum loop. Each experiment was performed with at least 90 worms. The data represent the mean of three independent experiments with three replications (*N* = 270).

### 4.5. Western Blot Analysis of Aβ Species in C. elegans

Worms were harvested in ddH_2_O containing 1× protease inhibitor cocktail (Sigma, Oakville, ON, Canada), flash frozen in to liquid nitrogen and stored at −80 °C, until use. For protein extraction, worms were boiled at 105 °C for 10 min in a lysis buffer (*i.e.*, 62 mM Tris-HCl pH 6.8, 2% SDS (w/v), 10% glycerol (v/v), 4% β-mercaptoethanol (v/v) and 1× protease inhibitor cocktail), and then cooled, on ice, and centrifuged for 5 min at 14,000 G at 4 °C. The protein in the supernatant was quantified using Bradford Reagent (Bio-Rad, Mississauga, ON, Canada). Electrophoresis and Western blot analysis was performed following a previously published method [34]. Equal loading was determined by replicated, non-transferred Coomassie Blue stained, SDS-PAGE gel. The data was expressed as the mean of 5 biological replicates, using 4 and 20 kD bands in the analysis.

### 4.6. In Vivo Measurement of ROS in C. elegans

Intracellular ROS were measured in *C. elegans* strain CL4176, using the 2′,7′-dichlorofluorescein diacetate (H2DCF-DA) method [35]. Briefly, freshly laid eggs (60–65 eggs per plate) were transferred to the control or CCE (1 mg/mL) amended NGM plates and incubated for 36 h at 16 °C. To initiate amyloid-induced, progressive paralysis, the worms were shifted to an incubator set at 23 °C. The worms were harvested 24 and 32 h after the temperature shift using 500 μL of phosphate-buffered saline (PBS), washed twice with PBS to remove *E. coli* (OP-50) cells and then transferred into 96-well plates (Costar) in 200 μL of PBS containing Tween 20 (0.01%) and H2DCF-DA (50 μM). In a parallel experiment to determine tolerance level during oxidative stress, juglone (300 μM) was added to the PBS-*C. elegans*-H2DCF-DA mixture before placing the plate for fluorescence detection, which was quantified in a Synergy HT micro-plate reader (Bio-Tek Instruments, Winooski, VT, USA) for 6 h at 37 °C, using excitation at 485 nm, and emission at 530 nm. The data presented here represented the mean ± SE of three independent experiments, expressed as percentage fluorescence, relative to the MeOH control.

### 4.7. Fluorescence Microscopy of Reporter Gene Expression in CCE-Treated C. elegans

In order to determine the stress response mechanisms in *C. elegans* that were induced by the CCE treatment, the expressions of *HSP-16.2*, *GST-4* and *SKN-1* were studied in *C. elegans* strains TJ375 (*hsp-16.2*/GFP), CL2166 (*gst-4*/GFP) and LD1 (skn-1/GFP), respectively. Synchronized TJ375 eggs were transferred on to NGM plates containing the extracts and incubated for 2 days at 20 °C. To induce the heat-shock response, L4 worms were shifted to 35 °C for 2 h, followed by recovery at 20 °C, before taking digital pictures. To determine the role of *gst-4* which encodes a glutathione-*S*-transferase and is a downstream effector of the conserved *skn-1*, phase II detoxification pathway [36], freshly laid eggs of CL2166 were transferred to NGM plates with CCE (1 mg/mL) and grown at 16 °C until the L4 stage. A sub-section of the treated worms were shifted to freshly prepared NGM containing 300 µM juglone for 1 h and their fluorescence was recorded. To determine the role of *SKN-1*, the *LD1* (skn-1/GFP) worms were reared on treatments as above. Pre-treated L4 worms were transferred to fresh NGM plates containing 100 µM juglone and incubated overnight before observed for fluorescence under a microscope.

For quantification of fluorescence, the worms were anesthetized with sodium azide (10 mM) on an agarose pad, on a glass slide and their fluorescence was recorded (Olympus, Japan) with excitations at 488 nm and emissions at 500–530 nm. The images were acquired with a CCD camera (Leica Microsystems, Richmond Hill, ON, Canada), and the intensity was analyzed using Image-J software. The experiments were repeated twice with 15–20 worms per group; data were presented as mean ± SE of two experiments.

### 4.8. Expression Analysis of Stress-Induced Genes in C. elegans

Quantitative Real Time-PCR was conducted on CL4176 worms treated with CCE *vs.* control in order to relate their phenotypic and biochemical responses with the molecular response, under conditions leading to expression of Aβ protein species in the worms. For this, the transcriptional response of β-amyloid transgene (*amy-1)*, stress induced transcription factor *(skn-1)*, superoxide dismutase 3 (*sod-3*) and small heat shock protein (*hsp-16.2*) was monitored. Eggs were transferred to treatment plates and incubated for 36 h at permissive (16 °C) temperatures before shifting to 23 °C to induce *amy1* expression. The worms were sampled at 20 h after temperature up-shift. The worms (80–100) were transferred directly into TRIzol Reagent (100 µL; Invitrogen Life Technologies, Burlington, ON, Canada) and flash frozen in liquid nitrogen. Total RNA was extracted with TRIzol reagent following standard protocol and cDNA was synthesized with a High Capacity cDNA Reverse Transcription kit (Applied Biosystems, Burlington, ON, Canada). The RT-PCR primers were as follows: *amy-1* (forward, 5′-CCGACATGACTCAGGATATGAAGT-3′; reverse, 5′-CACCATGAGTCCAATGATTGCA-3′); small heat shock protein *hsp-16.2* (forward, 5′-ACGCCAATTTGCTCCAGTCT-3′; reverse, 5′-GATGGCAAACTTTTGATCATTGTTA-3); *sod-3* (forward, 5′-AGCATCATGCCACCTACGTGA-3′; reverse, 5′-CACCACCATTGAATTTCAGCG-3′); and *skn-1* (forward, 5′-AGTGTCGGCGTTCCAGATTTC-3′; reverse, 5′-GTCGACGAATCTTGCGAATCA-3′). The gene *ama-1* (forward, 5′-CTGACCCAAAGAACACGGTGA-3′; reverse, 5′-TCCAATTCGATCCGAAGAAGC-3′) was used as the internal control. The transcript abundance was assessed using StepOne™ Real-Time PCR System (Applied Biosystems, Foster City, CA, USA) using an SYBR green reagent (Roche Diagnostics, Mississauga, ON, Canada). Data were analyzed from two independent runs (mean ± SE) and expressed as relative expression.

### 4.9. Chemical Profiling of CCE and Active Fractions

The NMR spectra were measured using a Bruker 500 or 700 MHz spectrometer with deuterated solvent. The CCE and CCW extracts (20 mg/mL) were dissolved in deuterated water containing 1 mM Sodium-(trimythylsilyl)-2,2′,3,3′-tetradeuratedpropionate (TMSP) and filtered through 4 mm, nylon 0.2 µm syringe filter (Canadian Life Science, Toronto, ON, Canada). Concentration of the major components present in the extracts and aqueous fraction were calculated based on the integration of individual protons as compared to the internal standard. The fatty acid profile of the active fraction was analyzed using LC/MS. Agilent 1200 Series HPLC coupled with 6100B Series Single Quadrupole LC/MS systems was used for LCMS analysis. The MGDG rich fraction (CC5) was subjected for LC/MS analysis using Synergi MAX-RP column (4 μm, 4.60 mm × 250 mm, Phenomenex, Torrance, CA, USA) with 95% MeOH/0.025M H_2_SO_4_ in H_2_O isocratic condition for 45 min at 1.0 mL/min.

### 4.10. Statistical Analyses

Statistical analyses were performed using JMP software (SAS institute Inc., Cary, NC, USA). One-way ANOVA was used for group comparisons using the Student’s *t* test. For β-amyloid-induced paralysis assays, the data were analyzed with Kaplan–Meier survival curves and the p values were calculated by log-rank comparison between the control and the treatments (* ≤ 0.05; ** < 0.0001).
